# Dr. John Jay Taylor

**Published:** 1912-09

**Authors:** 


### OBITUARIES.

---

Dr. John Jay Taylor of Philadelphia, Medico-Chirurgical, 1887, died at Ocean City, N. J., Aug. 1, after a long illness, aged 59. He was a member of the A. M. A. and its component local organizations and of the American Medical Editors' Association. Since graduation, he had devoted himself to medical literary work, at first on the staff of the *Medical World*, later as chief editor of the *Medical Council*. Our acquaintance began at Philadelphia in 1897, when we were associate editor of the old *Philadelphia Medical and Surgical Reporter* and our last meeting was at Atlantic City, in June, 1912. Dr. Taylor was a man who endeared himself to all.

